# Genetic Interference: Don’t Stand So Close to Me

**DOI:** 10.2174/138920210790886835

**Published:** 2010-04

**Authors:** Luke E Berchowitz, Gregory P Copenhaver

**Affiliations:** Department of Biology and the Carolina Center for Genome Sciences, University of North Carolina at Chapel Hill, Chapel Hill, North Carolina 27599-3280, USA

**Keywords:** Meiosis, recombination, crossover, Double-strand break, synaptonemal complex, chromosome Spo11, Rec8, Pch2.

## Abstract

Meiosis is a dynamic process during which chromosomes undergo condensation, pairing, crossing-over and disjunction. Stringent regulation of the distribution and quantity of meiotic crossovers is critical for proper chromosome segregation in many organisms. In humans, aberrant crossover placement and the failure to faithfully segregate meiotic chromosomes often results in severe genetic disorders such as Down syndrome and Edwards syndrome. In most sexually reproducing organisms, crossovers are more evenly spaced than would be expected from a random distribution. This phenomenon, termed interference, was first reported in the early 20^th^ century by Drosophila geneticists and has been subsequently observed in a vast range of organisms from yeasts to humans. Yet, many questions regarding the behavior and mechanism of interference remain poorly understood. In this review, we examine results new and old, from a wide range of organisms, to begin to understand the progress and remaining challenges to understanding the fundamental unanswered questions regarding genetic interference.

## BACKGROUND

Meiosis, a type of cell division, reduces the chromosomal complement by half to produce gametes that are essential for sexual reproduction. During meiotic prophase, chromosomes pair with their homologs and, in most organisms, undergo a physical exchange of DNA or an exchange of sequence information in a process called recombination [1]. Recombination is initiated by programmed double-strand breaks (DSBs) of chromosomes. During the repair of some DSBs, chromosome arms are exchanged generating crossovers (COs).

In most organisms, COs are not distributed randomly. Closely spaced COs are observed less frequently than would be expected from a random distribution. This phenomenon is known as crossover interference – though the more general term genetic interference may be more useful since there is growing evidence (described below) that other events can also interfere with one another. Alfred H. Sturtevant and Hermann J. Muller are typically given equal billing for the discovery of interference. Sturtevant clearly describes the phenomenon as early as 1913 [2]. Two years later, he coined the term “interference”, though in doing so he gives credit to Muller for suggesting the name and also for his influence in discovering the phenomenon [3]. For clarity, in this review ‘interference’ will refer to positive interference, which is the spacing of events that departs from a random distribution, as opposed to negative interference, which describes events that are more clustered than the null expectation. Although interference was originally observed almost a century ago and has subsequently been validated in numerous studies, fundamental questions regarding its underlying mechanisms still exist. The goal of this article is to outline unanswered questions about interference and also to review existing models.

The genomic distribution of COs is regulated in multiple ways. For example, they are distributed such that each chromosome typically receives at least one, which is known as ‘CO assurance’. A random distribution of COs among chromosomes predicts a class of chromosomes that have no COs, yet the observed number of chromosomes without a CO is quite small in most organisms [4, 5]. Growing evidence suggests that interference is the result of multiple levels of recombination regulation and CO assurance could be a result of the interference mechanism.

**Fig. (1):**
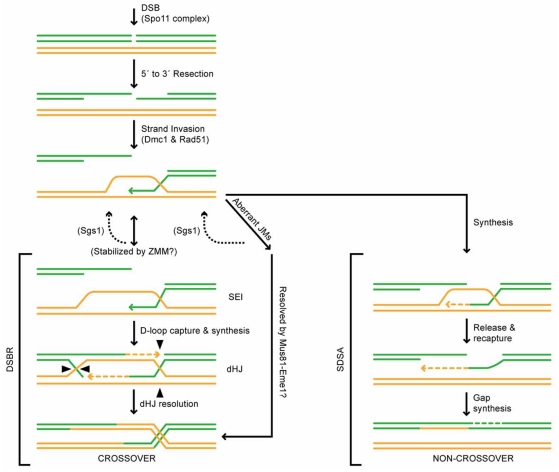
The DSBR and SDSA meiotic recombination models. Single strands of DNA are shown as either green (parent 1) or yellow (parent 2) rods. The Spo11 complex initiates programmed DSBs. DSBs are resected 5’ to 3’ to produce single ssDNA tails. ssDNA tails invade the homologous template which is aided by the ssDNA filament forming proteins Dmc1 and Rad51. At this stage, intermediates can undergo DSBR (left), which is thought to produce primarily COs or SDSA (right), which only produces NCOs. Also shown is a pathway (center) describing aberrant JMs, that are hypothesized to either be resolved back to the strand invasion stage by Sgs1 or resolved as COs by the Mus81-Eme1 heterodimer [52]. In the DSBR pathway, strand invasion complexes are stabilized (possibly by ZMM proteins) to form SEIs. Prior to stabilization, Sgs1 could wire SEIs back to the strand invasion stage [52, 89]. The displaced strand, called a D-loop is captured by the resected break of opposite homolog and subsequent DNA synthesis results in a dHJ intermediate. This intermediate is resolved as a CO upon appropriate resolution of the two HJs. in the SDSA pathway, the invading strand dissociates after a patch of DNA synthesis. This strand then re-anneals to the original parent, resulting in repair of the DSB and a patch of heteroduplex DNA. This pathway is always resolved as a NCO, but it can result in GC.

Meiotic DSBs are enzymatically catalyzed by a topoisomerase I-like protein called Spo11 that remains covalently attached to 5' ends of the break (Fig. **1**). Following Spo11 removal and further end processing (resection), the breaks are left with single-stranded 3' tails. One of these tails can then invade a non-sister chromatid, which is known as strand invasion. Stabilized strand invasion intermediates are known as single-end invasion (SEI) intermediates. The free 3'-OH in the SEI structure is used as a substrate by DNA polymerase to extend the 3' tail and the size of the displaced DNA strand (D-loop). The DNA synthesis that occurs at this stage is primed by one chromatid but uses a non-sister chromatid as a template – therefore any polymorphisms that exist at this locus will be copied from the template chromatid to the invading chromatid. This transfer of parental information is called gene conversion (GC) [6, 7]. At this point the invading end can dissociate from the non-sister chromatid and re-associate with the other end of the break in a process called synthesis dependant strand annealing (SDSA) [8]. After additional DNA synthesis and ligation the break is repaired resulting in a non-crossover (NCO), potentially with associated GC if a polymorphism existed at the locus. It is important to note however that GC would result in heteroduplex DNA – i.e. that Watson and Crick strands of the converted chromatid would have non-complementary bases at the polymorphic site. The heteroduplex DNA can be recognized by the cell’s mismatch repair system and either repaired such that the original parental genotype is restored or such that the converted genotype is kept. Alternatively, the mismatch repair system can fail to recognize the mismatch. In this case, both genotypes in the heteroduplex will be propagated during the next mitotic division in a process called post-meiotic segregation (PMS). PMS results in two populations of cells, one that has experienced GC at the polymorphic site and the other that has not.

If SDSA does not occur, then as the D-loop is extended it can hybridize to the single-stranded 3' tail on the other side of the break in a process called second end capture (Fig. **1**). Again, this structure can be acted on by DNA polymerase, which extends the second single-stranded 3' tail. As before, the priming and template DNA are from non-sister chromatids, therefore GC can occur during this stage. Following DNA synthesis and subsequent ligation, an intermediate called a double Holiday junction (dHJ) is formed. In principle, this structure can be resolved to produce either a crossover (CO) or NCO depending on how the individual junctions are cut to release the chromatids. However, evidence in *Saccharomyces cerevisiae* suggests that these intermediates are resolved predominantly as COs [4, 9, 10]. In either case, the resulting COs and NCOs can be associated with GC.

### Early Observations

Sturtevant observed that in *Drosophila melanogaster* a three-point cross involving mutations on the X chromosome (yellow, white and miniature wings), a CO in the yellow-white interval occurred at a frequency of 1/69 gametes without the presence of a neighboring CO in white-miniature, but this frequency plummeted to 1/441 when there was a CO in the white-miniature interval (P = ~0.25) [3]. Sturtevant went on to define an “index of interference” as the expected probability of a double CO (the product of the two individual recombination frequencies) divided by the observed frequency of double COs. This marked the beginning of the debate as to the best way to measure interference, which is still being argued to this day [11]. With the index of interference and additional data Sturtevant decreed that “(interference) is less when the intervals are larger and vice versa [3].”

Data generated in Drosophila dominated advancements in interference research for the next 40 years [12-15]. Subsequent papers lacked empirical observations that changed the essential view of interference; instead they confirmed Sturtevant’s initial observations and focused on different ways to measure interference. Nonetheless, several key observations were made. Muller first proposed that interference does not act between different chromosomes, i.e. a CO on one chromosome does not affect the probability of a simultaneous CO in an interval on another chromosome [13]. Subsequent research by Weinstein established the reach of interference on the X chromosome. In this case, interference does not manifest when the distance between the two intervals being studied is greater than 46 cM [14]. Additionally, COs in intervals on the same chromosomal arm interfere more strongly than intervals on opposite arms that have approximately equivalent genetic distances [14]. This introduced the idea that interference does not cross the centromere. In 1932, Graubard observed that chromosome 2 carrying an inversion (max size 25 cM) did not affect interference values of intervals on that chromosome [15]. This result was the first to suggest that pre-existing chromosomal features are not the primary determinant of interference, which is widely believed to this day.

Model fungal organisms that form tetrads (fused meiotic products), allow for both the recovery of non-reciprocal recombination products and novel statistical approaches for interference analysis [16-18]. Non-Mendelian 6:2 segregation (the result of post-meiotic mitotic division of a 3:1 tetrad) at a single locus, indicative of a GC, was first observed in spore pigmentation mutants of *Bombardia lunata* [18]. These observations led researchers to question if GCs were born from the same mechanism that produces COs. If true, this would predict that they should exhibit interference. Using *Neurospora crassa* as a model system, Stadler tested this idea by seeing if GC events interfered with the probability of COs in an adjacent interval. He found that GCs did not interfere with COs and (errantly) concluded that GCs and COs arose from different precursors [19]. Subsequent research by Mortimer and Fogel demonstrated that GCs occur with or without flanking marker exchange establishing the idea of NCO and CO repair [20]. These data suggested that all recombination events have a common molecular basis (which we now know to be DSBs and strand invasion [6, 7]) and that initial events are distributed independently of one another, but that the occurrence of a CO at one site will subsequently influence nearby events to be resolved as NCOs, thus resulting in a CO distribution that displays interference [20].

## UNANSWERED QUESTIONS

### What Types of Events Interfere with One Another?

Interference is often referred to as ‘CO interference’, which does not capture the breadth of the phenomenon, since COs may not be the only recombination-related events to inhibit one another’s distribution. Determining what combinations of recombination events are subject to interference is key to understanding both when interference is imposed as well as the mechanism that mediates it.

### DSB-DSB Interference

Interestingly, in many organisms, not even all COs are subject to interference (discussed below). However, those COs that do interfere could theoretically be a reflection of an inhibition of closely spaced DSBs. Whether or not, and to what degree DSBs interfere with one another remains an open question. Meiotic DSB mapping studies in *S. cerevisiae* have resulted in a detailed understanding of where DSBs are most likely to occur [21-24], but they are not ideal for addressing whether or not DSBs are subject to interference because DSB mapping is an amalgamation of thousands of independent meioses so even if the DSBs in individual meioses were subject to interference, it would likely be obscured by the layering of data. It may be relevant that the hottest DSB hotspots only have a break in ~10% of meioses [21] and periodicity in DSB hotspot distribution has never been reported. However, in *S. cerevisiae*, researchers have clearly shown competitive DSB inactivation whereby insertion of a strong DSB hotspot reduces the frequency of DSB formation and recombination in nearby regions [25, 26]. Ohta *et al.* have reported that insertion of an artificial DSB hotspot results in a parallel decrease of DSBs in a ~60 kb region around the insertion site [25]. This led to the proposal that strong DSBs sites could outcompete nearby sites for limiting factors essential for DSB formation. Another proposal is that structural features that influence DSB formation create domain boundaries that isolate regions from one another [25].

DSB distribution has also been addressed *via *mapping the physical position of structures called recombination nodules (RNs). RNs are proteinaceous structures that are associated with the axial elements of the synaptonemal complex (SC) from leptotene to pachytene and are thought to be locations where meiotic recombination reactions are occurring [27]. RNs are divided into two sub-classes: early nodules (ENs) and late nodules (LNs), which differ in respect to timing, size, shape and number. ENs, the smaller of the two, roughly correspond to Rad51/Dmc1 foci [28], while LNs, which most likely arise from a fraction of ENs, are thought to be the molecular machinery that execute COs and thus represent CO sites [27, 29]. In tomato, both ENs and LNs exhibit a distribution indicative of interference, but the strength of interference is much stronger among LNs [30, 31].

Analysis of the distribution of *MSH4* foci in mouse meiocytes strongly supports the idea that DSBs interfere with one another. In zygotene, ~150 *MSH4* foci are initially detected, which reduces to ~50 foci by late pachytene. Early *MSH4* foci co-localize with *RAD51*/*DMC1* and are thought to mark all DSBs in the early repair stages, while late *MSH4* foci co-localize with *MLH1* foci and are thought to mark CO sites only [32-34]. Early *MSH4* foci exhibit a distribution indicative of interference, but do not exhibit as strong interference as *MLH1* foci [31, 35, 36].

Taken together the above results argue that interference is the result of multiple layers of control [31, 32, 36]. DSBs may exhibit positive interference over short distances, but DSB-DSB competitive interaction would not be sufficient to explain CO interference over megabase (Mb) distances.

### NCO-NCO and NCO-CO Interference

Another observation that suggests interference is not merely a reflection of an underrepresentation of closely spaced DSBs is that in *S. cerevisiae*, NCOs do not interfere with other NCOs. Mortimer and Fogel reported that GC events at *ARG4* and *THR1* (~19 kb apart) do not interfere with one another since alleles at the two loci co-converted at a frequency that is indistinguishable from independence predicted by their individual conversion frequencies. More recently, analysis of genome-wide recombination maps based on DNA tiling arrays reaffirmed that the distance between GCs not associated with COs does not differ significantly between experimental samples and randomized control data [37].

Whether or not COs interfere with NCOs (or vice versa) is a more controversial topic. The first results to address this issue came from *S. cerevisiae* when Mortimer and Fogel showed that a GC at *HIS1* or *ARG4* with exchange of flanking markers (CO) results in a decrease of genetic distance in an adjacent genetic interval, whereas a GC without exchange of flanking markers (NCO) actually promoted CO frequency in the adjacent interval (negative interference) [20]. The idea that NCOs do not interfere with COs was supported by Malkova *et al*. who showed that, at the *met13* locus of *S. cerevisiae*, GCs without an accompanying CO did not exert positive interference on adjacent intervals, while GCs associated with a CO did [38]. However, they did not observe statistically significant negative interference between GCs not associated with CO at *met13 *and COs. Recently, Getz *et al*. showed that NCO GCs did not exert interference on adjacent intervals and the map distances in those intervals were actually increased indicative of negative interference, in support of the idea that NCOs do not positively interfere with COs [39]. However, most recently, a contradictory result showing that NCOs and COs interfere with one another was recently presented by Mancera *et al.* who, using the genome-tiling method, showed that inter-event distance between NCO and CO events were on average ~13kb larger than expected from a random distribution, representing statistically significant, albeit weak, positive interference [37]. While locus-by-locus studies have consistently shown negative or no interference between NCOs and COs, this genome-wide approach showed the opposite. It is difficult to reconcile these contradictory results, but because the Mancera data were generated using a genome-wide analysis the advantage appears to be with the idea that COs and NCOs exhibit positive interference. Nonetheless, it is interesting to note that models in which DSBs that are resolved as COs influence nearby DSBs to be resolved as NCOs predict negative interference between COs and NCOs. One reconciliatory possibility is that there exist both interfering NCOs and non-interfering NCOs (as in COs, see below) and the single locus studies mentioned above happened to measure only the latter class.

### CO-CO Interference and Non-Interfering COs

In many organisms, there are at least two pathways for producing COs. *Arabidopsis thaliana*, humans, mouse and *S. cerevisiae* have one pathway constituting the majority of COs that is sensitive to interference and a secondary pathway that produces interference-insensitive (randomly distributed) COs [40]. In these organisms, primary pathway COs are characterized by the Msh4-Msh5 heterodimer while secondary pathway COs are dependent on the Mus81-Eme1 heterodimer [40]. In *S. cerevisiae*, *msh4Δ* or *msh5Δ *deletions have a ~60% reduction in COs and the remaining COs are interference insensitive [41-43], while *mus81Δ* or *mms4Δ* (*eme1*) deletions have a ~25% reduction in COs and the remaining COs are sensitive to interference [40, 41]. In Arabidopsis, an analogous situation exists where interference-sensitive COs mediated by the Msh4-Msh5 pathway make up ~80-85% of the total while interference insensitive COs mediated by the Mus81-Eme1 pathway make up ~15-20% of the total [44-47]. Not all organisms produce both interfering and non-interfering COs. *Schizosaccharomyces* *pombe* does not have CO interference and ~80-95% of its COs are dependent on Mus81-Eme1 [48, 49]. In contrast, *Caenorhabditis elegans* has ‘perfect’ interference (exactly one CO per bivalent) and all COs are dependent on Msh4-Msh5 [50]. *D. melanogaster* is not thought to produce interference-insensitive COs and is thought to exhibit absolute interference across distances < ~10 cM [51]. A comparison of interference patterns in different organisms is presented in Table **1**.

While it is known that both interfering and non-interfering COs can be produced in the same cell, the mechanistic difference between the two and specifically why one class exhibits interference and the other does not is unknown. One possibility is based on the work in Arabidopsis by Franklin *et al. *in which they found that, during leptotene, ~115 *AtMUS81* foci form on chromosome axes and many of these co-localize with *AtRAD51* and *AtMSH4* foci, each of which form 80-100 foci during leptotene/early zygotene [47]. By early pachytene, the number of *AtMUS81* foci drops precipitously to ~5. This suggests a ‘toolbox hypothesis’ where Mus81-Eme1 and Msh4-Msh5 are recruited to all DSBs and most are repaired *via *the Msh4-Msh5 pathway, while Mus81-Eme1 acts to resolve a subset that may consist primarily of aberrant joint molecules (JMs) as either COs or NCOs that could not be repaired using Msh4-Msh5. This could result in interfering and non-interfering COs (and perhaps non-interfering NCOs) if the Msh4-Msh5 pathway is subject to interference, but the smaller and randomly distributed population of aberrant JMs resolved as COs by Mus81-Eme1 acts later, after interference has already been established. In congruence with this idea, recent biochemical and genetic analysis of *mus81 *mutants in *S. cerevisiae* indicates that Mus81-Eme1 acts late in meiotic recombination and likely resolves aberrant JMs that cannot be resolved by the primary Msh4-Msh5 pathway [52, 53] (Fig. **1**). Another related possibility is that primary CO reactions occur first and initiate an ‘interference signal’ (discussed below), while secondary COs take longer to process, as they must first be bypassed by the primary pathway. In this model, secondary COs do not produce an interference signal, and are resolved after the interference signal has been imposed.

An alternate hypothesis regarding the difference between interfering and non-interfering COs was introduced by Getz *et al*., who proposes two phases of COs. Early ‘pairing phase’ COs are non-interfering and late ‘disjunction phase’ COs, that are dependent on *MSH4*, exhibit positive interference [39]. In addition to being independent of *MSH4*, pairing phase COs are hypothesized to be less proficient at repairing mismatches. The hypothesized existence of MSH4-independent ‘pairing phase’ COs in *S. cerevisiae* is consistent with the lack of non-interfering (Mus81-dependent) COs in *C. elegans* and Drosophila, because these organisms do not use COs to pair their chromosomes [54-56]. This model is also compatible with the phenotype of *ndj1* mutants, which have decreased interference and increased rates of nondisjunction [57, 58], which can be explained by increased pairing COs at the expense of disjunction phase COs. Since pairing phase COs are proposed to be interference insensitive it follows that they should be *MUS81*-dependent, which predicts that *mus81* and *eme1* mutants will be pairing defective. In yet another layer of complexity the toolbox and two-phase hypotheses are not mutually exclusive as there could be pairing phase non-interfering COs that have nothing to do with *MUS81* as well as non-interfering disjunction phase COs produced by *MUS81* that are reflective of *MSH4*-independent aberrant JM resolution.

### What is the Timing of Events Leading to Interference?

Determining the timing of events that lead to interference is extremely challenging since diverse cellular processes likely play a role. Chromatin structure (e.g. nucleosome density, protein-DNA complexes, histone modifications etc.) and steric features of the chromosomes could influence recombination complex spacing. These features are not constant along chromosomes and are dynamic in both the mitotic and meiotic cell cycles. Meiotic chromosome condensation, which begins at the start of meiotic prophase and does not end until after recombination is complete, also likely influences interference [1, 29, 59]. However, pre-recombination chromosomal features are not the only important determinants. In many organisms, mutations in the meiosis-specific ZMM (*ZIP*, *MSH*, *MER*) recombination genes, which act after SEI formation, result in the abolition of interference. This strongly suggests that the assembly and distribution of recombination complexes is critical for the timing of the imposition of interference.

One attractive proposal is that interference is imposed during strand invasion when Msh4-Msh5 complexes stabilize CO-specific (SEI) recombination intermediates [60]. This idea is based on the observations that *msh5 ndt80* mutants result in very low levels of JM accumulation along with absence of interference in *S. cerevisiae* [61], but *spo16 ndt80 *mutants, which are defective for synaptonemal complex (SC) extension, result in high JM accumulation but wild-type interference [60]. The *ndt80* mutation was used in this case because it removes the late pachytene checkpoint and results in accumulation of recombination intermediates [60]. The *spo16* mutant also offers important insight as to the latest interference could be acting. Because *spo16* mutants are defective for SC extension and yet have wild-type interference it is likely that interference is fully implemented before late leptotene/early zygotene when the SC is formed [1]. Supporting this idea is the observation that SC initiation complexes exhibit a distribution indicative of interference [62]. Additional support for the idea that interference involves regulation of the strand invasion step comes from analysis of the *tid1* mutant in *S. cerevisiae* [63]. Tid1 is an accessory factor that facilitates strand invasion and the *tid1* mutant displays ~wild-type levels of COs yet interference is significantly weakened [63].

The timing of events leading to interference is different in Drosophila and *C. elegans*, in which pairing and synapsis occurs prior to the initiation of recombination. Thus, interference in these organisms is likely implemented after (though not dependent on) SC formation.

### Do COs Influence Nearby DSBs to be Repaired as NCOs? 

Analysis of the ZMM mutant phenotypes has led to the early decision model, in which the commitment of a DSB to be repaired as either a NCO or CO is made at, or prior to, stable SEI formation [10, 64]. How this decision is enforced is unknown. ZMM mutants are strongly CO defective, have abolished or greatly reduced interference, yet are not defective for NCO formation [42, 65, 66]. 2-D gel analysis of DNA intermediates has shown that the kinetics of DSB and NCO repair are normal in ZMM mutant backgrounds, but stable SEI production is inhibited. This observation strongly suggests that ZMM genes are not required for designation of DSB repair as either COs or NCOs and that this designation is made prior to SEI formation [10, 64]. NCO/CO designation is a key determinant of CO distribution and thus interference. It is important to determine if designation of a CO at one site subsequently results in NCO designation of nearby DSBs, which is an attractive, but as yet unproven, proposal. The other possibility is that all DSBs are designated as either COs or NCOs independently of one another in a distribution reflective of CO interference.

### What is the Role of *PCH2* in the Mediation of CO Interference? 

Recent mutant analyses strongly implicate the AAA+ ATPase Pch2 as an important regulator of CO interference, however the pathway by which Pch2 mediates interference is unknown. Two independent studies in *S. cerevisiae* showed that *pch2∆* mutants exhibit significantly weakened interference at several loci [67, 68]. Interestingly, both groups reported no significant changes in CO frequency on chromosome III (the shortest *S. cerevisiae* chromosome) indicating that the processes of CO formation and CO interference can be decoupled, at least on short chromosomes. However, the two studies presented incongruent results regarding CO frequency on other chromosomes in that Joshi *et al*. report no significant changes in CO frequency at any loci [67] while Zanders and Alani report significant increases in CO frequency on medium and large chromosomes [68].

*pch2∆* mutants display elevated Zip3 foci (an early marker of CO designated sites), aberrantly diffuse Hop1 localization and increased SC length [67]. In light of these findings, the researchers propose a short-range interference model (discussed further below) in which Pch2 aids in the establishment of chromosomal domains in which only one CO can occur [67]. In addition to increased CO frequency, *pch2∆* mutants display no increase in DSBs and exhibit elevated CO:NCO ratios at two GC loci, compared to wild-type [68]. Importantly, the researchers rule out excess COs as the explanation for weakened interference in *pch2∆* since *pch2∆; *Spo11-hypomorph double mutants display ~74% of the COs of *pch2∆* while maintaining the interference defect. Additionally, *pch2∆*; *mms4* double mutants have significantly higher CO frequency than *mms4* mutants, which one would not see if the interference defect and CO frequency increase was solely due to elevated secondary pathway (Mus81-Mms4 dependent) COs in *pch2∆* [68]. To explain the *pch2∆ *phenotypes of increased CO frequency, increased CO;NCO ratio and weakened interference, the researchers propose that Pch2 acts to repress the CO designation at the CO/NCO bifurcation in the decision pathway [68]. While *PCH2* very likely plays an important role in interference, this gene is a piece in the puzzle since a) the interference defect in *pch2∆* is not present at all loci that display interference [67] b) *pch2∆* mutants have residual positive interference [67, 68] and c) the interference defect in is most prominent in 50-100kb distances and can be mitigated at lower temperatures [67].

### Do Meiosis-Specific Cohesins Mediate Interference?

In *S. cerevisiae*, the meiotic cohesin complex consists of Scc3, Smc1, Smc3 and Rec8, all which are also present in the mitotic cohesin complex except Rec8 [69, 70]. In addition to its role in sister chromatid cohesion, Rec8 has been implicated in a diverse set of meiotic processes including pairing, SC polymerization, recombination, and disjunction [70, 71]. Many of these functions have been shown to be separable from its role in sister chromatid cohesion [71]. In *S. cerevisiae* and *S. pombe*, Rec8 has a role in the binding of Spo11 to DSB sites in a region-specific manner [72, 73]. ChIP-chip reveals an interesting correlation between binding of Spo11 and cohesins. Spo11 has been shown to co-localize with Rec8 in early meiosis, and the frequency of co-localization decreases as meiosis progresses [73]. It has been proposed that Rec8 provides structural landmarks that dictate the proper distribution of Spo11 [73]. Spo11 could transfer from Rec8 binding sites to chromatin loops before initiating DSBs [73]. Since Rec8 has a role in dictating DSB distribution, a role in the establishment of the weak DSB-DSB interference discussed earlier is possible. The Rec8 binding landscape could serve to bias Spo11 distribution toward uniformity. However, a role for Rec8 in the mediation of interference has not yet been demonstrated, so additional work to elucidate this relationship will have to be conducted.

## MODELS

The central unanswered question regarding interference is how it is achieved within the cell. The answer to this question has been elusive partly because traditional genetic screens designed to discover interference mutants are labor-intensive and problematic. Isolating the interference machinery is difficult because the relevant players likely have overlapping roles with other critical meiotic processes. Most mutations that affect interference also affect CO frequency and only a handful of mutants has been identified (all in *S. cerevisiae*) that exhibit an interference defect without a concurrent hypo- or hyper-CO phenotype [57, 63, 67]. Further complicating matters is that many of these mutants behave differently with regards to CO frequency in different studies and within studies at different loci [39, 57, 63, 67, 68, 74]. Additional complications arise from the fact that interference is not absolute in most species but instead reflects the reduced probability of an event in a population of events – making the implementation of efficient screens difficult. Models aimed at explaining the interference mechanism have been proposed, but there is no dominant paradigm as no empirical observations vastly favor one particular explanation. Several commonly referenced models are discussed below.

### Mechanical Stress Model

**Fig. (2):**
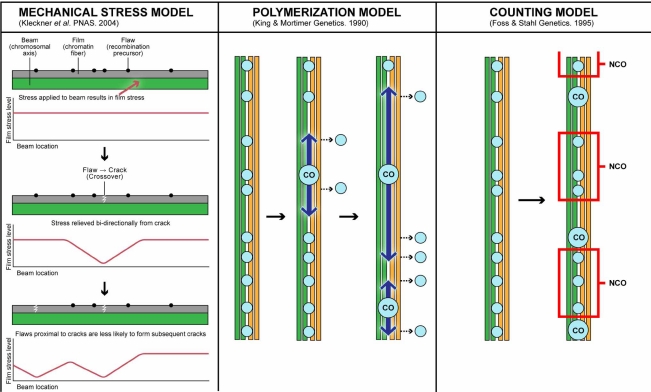
Interference Models. The left panel depicts the beam-film demonstration of the mechanical stress model proposed by Kleckner *et al*. [59]. The beam (chromosomal axis; green), film (chromatin fiber; grey), flaws (CO precursors; black dots). Diagrams depicting the stress level are shown under each beam in which the x axis represents beam position and stress level on the y. The center panel depicts the polymerization model proposed by King and Mortimer [75]. Chromatids are shown in green (parent 1) and yellow (parent 2). Small light blue circles represent recombination precursors and CO designates are shown as larger circles marked with ‘CO’. The interference polymer is shown as a large arrow emanating from CO sites, and CO precursors removed by the polymer are shown to the right accompanied with a dashed arrow. The right panel depicts the counting model proposed by Foss *et al*. [78]. Chromatids are shown in green (parent 1) and yellow (parent 2). Small light blue circles represent recombination precursors and CO designates are shown as larger circles marked with ‘CO’. In this diagram, m=3 and intervening NCOs between COs are outlined in a red box.

Because interference is maximized at close distances and decreases with increasing distance [3], interference models that rely on a mechanical explanation invoke a signal that spreads from CO sites. Muller’s original interference model posited that the stiffness of a chromosome would make it difficult to bend back on itself after a CO to form another in close proximity [13]. The modern mechanical stress model proposed by Kleckner *et al*., is based on the idea that in many physical systems, any increase or decrease in stress starts at a locus and propagates outward from that point (Fig. **2**) [59]. Stress is generated as meiotic chromosomes compress and expand. COs result in a localized relief of this stress that spreads in both directions down the axis of the SC. This model is attractive because it posits a simple explanation that predicts many properties of interference including CO assurance and CO homeostasis (discussed below). As each chromosome will be under stress, the occurrence of a first event, CO assurance, is easily obtained. Each event defines a domain of inhibition resulting from stress relief spreading from a CO, which acts as an inhibitory signal. Chromosomal features such as centromeres could act as sinks that absorb the signal, which would explain how interference does not cross the centromere. Multiple events could occur on the same chromosome, and could take place in different locations in different nuclei, but would always have the tendency to be evenly spaced. A mathematical simulation of this model has been used to fit CO data from two species [59]. However, the stress model does not make easily testable predictions. Additionally, it is easy to imagine how DSBs would relieve tensile stress, but less so for COs, which would be necessary for this model to explain all aspects of interference. Furthermore, it is also difficult to account for how some COs could mediate stress relief while others don’t which would be necessary to explain the data in organisms that appear to have both interfering and non-interfering crossovers.

An additional layer to the mechanical stress model was put forward to explain the role of Pch2 in the mediation of short-range (< 100 kb) CO-interference [67]. Joshi *et al*. propose a one-CO module hypothesis in which Pch2 aids in the establishment of domains that tile chromosomes and incur one and only one CO per module [67]. Mechanical stress within each module promotes a single CO that that relieves stress within the module. Key features of one-CO modules, presumably mediated by Pch2, are a centrally located Zip3 focus along with Hop1 hyper-abundance that extends to the edges of the domain, establishing the reaches of short-range interference [67]. The hypothesized contents of the modules are based on the observation that *pch2∆* mutants, along with weakened interference, display diffuse rather than domainal Hop1 staining and aberrant increased Zip3 foci [67]. The one-CO module model is capable of explaining interference across organisms with larger or smaller interference reach by varying the size of modules. For example, in *C. elegans*, which incurs exactly one CO per bivalent, each chromosome could be encompassed in entirety by one module. The one-CO module model does not explain interference over >100 kb distances in *S. cerevisiae *since in the pch2∆ mutant, where module establishment is presumed to be impaired, interference is unaffected in distances over 100 kb.

### Polymerization Model

The polymerization model describes a situation where early recombination structures are distributed independently of one another and then have an equal chance per unit time of initiating a bi-directional polymerization event (Fig. **2**) [75]. This polymer spreads from the site of initiation and has the ability to block additional early structures from binding to the bivalent. Sites of initiation are hypothesized to mature into LNs (COs) leading to chiasmata. This model is attractive because it explains interference and assurance while predicting a pattern in which interference is strongest nearest to initiation events with decreasing strength in a distance-dependant manner. A computerized simulation of the parameters described in the polymerization model was fit satisfactorily to CO data from *Drosophila* and *S. cerevisiae *[75].

Part of the rationale behind the polymerization model was that an optimal interference model should be useful in systems that differ by several orders of magnitude in genome size in bp. Physical distances measured in SC lengths rather than bp are much closer among species, thus the polymer is proposed to move down the axis of the SC. The idea that interference mediated over physical distance would be measured in SC length and that the interference signal is propagated along the SC axis is attractive because organisms of vastly different size genomes can be normalized by modifying the size of DNA loops that are associated with the SC axis. The main obstacle to the polymerization model is that the polymer itself has neither been identified nor observed. However, the search for a polymer as the signal may be a red herring as the signal could be a modification such as phosphorylation, methylation, acetylation or ubiquitination of a protein such as a histone or cohesin. SC polymerization itself has been proposed as an attractive mediator of interference [76, 77]. This would have demonstrated a clear role for the SC while being consistent with the observation that *S. pombe* lacks both SC and interference. However, the possibility that the SC is required for interference is extremely unlikely since in *S. cerevisiae* SC extension has been shown to be dispensable for interference [60] and in mouse, SC defective mutants have normal interference [31]. Lastly, in refutation of the idea that the SC is in any way required for interference, is that in Drosophila and *C. elegans* the SC seems to be complete before DSBs are formed.

### Counting Model

The counting model is a mathematical construct in which COs are separated by a fixed number (*m*) of intervening NCO events (Fig. **2**) [78]. It was proposed in part to reconcile the fact that, in terms of physical distance (bp of DNA or µm of SC), strength of interference varies by several orders of magnitude from organism to organism [78, 79]. The counting model can account for vast differences in genome size, as interference is dictated by genetic distance i.e. the initial density of precursors and the number of intervening events between COs. CO data from *Drosophila* and *N. crassa *fit the counting model predictions extremely well [78], but initially it was less successful at modeling CO distributions in *S. cerevisiae* and humans [79]. Additionally, *m* appeared to vary in the same organism between sexes and chromosomes [80]. Subsequently, a modified version of the counting model that allows a number of non-interfering COs (v) was suggested. This additional parameter allowed the counting model to satisfactorily fit CO data from *S. cerevisiae* [81], *A. thaliana *[45, 82], and humans [80] and in each of these systems, it has been shown that non-interfering COs exist [40, 44, 83].

Proposed biological equivalents for each parameter in the counting model include DSBs as precursors and NCOs as intervening events (*m*), however, the notion that DSBs are what is being ‘counted’ is not supported by subsequent observations. A prediction of the counting model when DSBs are ‘counted’ is that when the overall number of DSBs are reduced, COs and NCOs should reduce proportionally. It then follows that larger distances between COs should result. However, recent results failed to meet this expectation [84]. Cells appear to have a mechanism (called CO homeostasis) that ensures a certain number of COs per meiosis even if the pool of DSBs from which they arise is reduced. A series of *spo11* hypomorphic mutants that produce DSBs in decreasing frequencies compared to wild-type do not show proportional reductions in COs [84]. Instead, these mutants maintain CO levels at the expense of NCOs, which are reduced proportionally to the reduction in DSBs. CO interference is maintained at wild-type levels in all *spo11* hypomorphic backgrounds [84]. CO homeostasis is seen as a major obstacle to the counting model. However, if DSBs were not what is counted, but instead a factor that establishes DSB sites, the counting model could still be possible. Despite its clear predictions and modeling support, the counting model suffers from an absence of both *in vivo* evidence as well as a testable molecular mechanism for its execution.

### Other Models

Interference models can be described in terms of point-process in which precursors i.e. ‘points’ are distributed according to a mathematical function and then COs are ‘processed’ from these points according to a second mathematical function [85]. The ‘hard-core’ model is a point-process variant where points are dispersed in a Poisson distribution with a minimum physical distance between any two [85]. At each point, chromatids have a 50% chance of being involved in a CO. The hard-core model is much like a situation where DSB-DSB interference is the driver of CO interference, which current data strongly suggests is not the case (discussed above). This model does not conform particularly well with CO data from *Drosophila *[85].

Another interesting proposal involves a ‘chiasma determining mechanism’ that moves along the bivalent [78, 86]. This mechanism is hypothesized to move along the bivalent at a constant rate occasionally firing and thus determining CO sites that mature into chiasmata. After firing, the mechanism requires time to recharge while still moving, resulting in CO interference [86].

## CONCLUSIONS

### How is Interference Imposed?

Interference modeling has traditionally been concerned with the synthesis of models capable of explaining CO interference across many organisms with vastly different genome sizes and recombination rates. Beyond this, accurate models must be able to reconcile additional complex properties of interference such as multi-level control, non-interfering COs and early decision. Even what falls under the purview of the term interference is currently in question. In addition to the strong likelihood that recombination events other than COs interfere, it is also possible that both CO homeostasis and CO assurance could be products of an over-arching CO control mechanism that results in the lack of closely spaced COs. While the above models can be altered to account for these complexities, the truth likely lies in combination of the ideas that have been proposed to explain interference.

Since a growing body of evidence supports the idea that interference is imposed at multiple levels, a comprehensive interference model will account for the presence of DSB-DSB interference, CO-CO interference and possibly CONCO interference. Although it remains unclear whether or not and to what degree DSBs interfere with one another, it is possible that DSB-DSB interference acts on a smaller scale and is mediated by multiple factors. First, the pattern of Spo11 distribution may be established by the meiosis-specific cohesin subunit Rec8. Rec8 has been shown to colocalize with Spo11 in *S. cerevisiae*, and *rec8∆* mutants exhibit a drastic alteration in Spo11 distribution [73]. It has been speculated that Rec8 not only provides landmarks along the chromosomal axes that guide the distribution of Spo11, but it is also responsible for the transition of Spo11 from the axes to the loops, where breaks are subsequently formed [73] Rec8 could preferentially bind certain locations within the chromatin context of the chromosomal axis with intervening DNA loops, resulting in a minimum physical distance between subunits. This idea requires that only one DSB could be formed at each site of Rec8 localization, which would result in the minimization of closely spaced DSBs thus establishing a pattern of DSB-DSB interference on a small scale. Secondly, DSBs could recruit limiting break forming factors away from nearby sites [25].

In addition to small-scale DSB-DSB interference, we are intrigued by the possibility that CO-CO interference in *S. cerevisiae* and *A. thaliana* is mediated by an ‘interference signal’ that is initiated by the stabilization of SEIs by ZMM proteins. This hypothetical interference signal propagates either down the SC axial elements or the cohesin axis. A signal propagated down the axis rather than the DNA itself is attractive because it allows interference to act over very large distances of linear DNA by varying the DNA bp loop/axis ratio. One-CO modules can be incorporated as domains established on the chromosomal axes that create favorable conditions for signal propagation. Importantly, the SC itself is not required for interference in this model. Additionally, if we suppose that the amount of DNA traveled by the signal is dependent on the condensation of the chromosome, it could explain the ‘lack’ of interference in early pairing phase COs and ‘presence’ of interference in disjunction phase COs. COs could occur at various time points in the meiotic program and thus over a large range of states of chromosome condensation. The earlier a (interference-sensitive) CO is designated, the less it will interfere (and vice versa) due to the continual condensation of the chromosomes during the meiotic program. This idea predicts that if CO formation can be in some way be delayed, interference will strengthen, a hypothesis that can be most effectively tested in an organism (such as Drosophila), which has a single interference-sensitive CO pathway.

What is the nature of the interference signal? An intriguing and testable explanation is that a protein modification that is propagated from the majority of COs results in the inhibition of primary-pathway crossing over at sites where this modification spreads. The modified protein(s) could include axial elements, cohesins, or histones. This idea predicts that CO-CO interference can be largely eliminated by a mutation that blocks the spread of the inhibitory modification *via *the receiver or the modifier. Rec8 is a particularly interesting target since phosphorylation of Rec8 is important for many of its meiotic roles. Two *rec8* mutants with mutations at multiple phosphorylation sites exhibit disrupted synapsis and have delayed production of mature recombinants [71]. Since Rec8 phosphorylation is required for recombination in *S. cerevisiae*, the protein modification could be a dephosphorylation of Rec8 that disables crossing over. A second possibility is de-methylation of H3K4me3, which has been shown to mark sites of meiotic recombination [87]. Thirdly, modification of axial-element protein Hop1, which is localized in discrete hyper-abundant domains on zygotene chromosomes, is an interesting possibility. *pch2∆ *mutants abolish domainal localization of Hop1 while concurrently weakening interference [67].

An alternate model for the interference signal is based on the recent observation that CO hotspots are significantly correlated with DNA methylation [88]. This group proposed that areas undergoing recombination could be secondarily methylated, which could result in the inhibition of further COs in that area [88]. Another possibility is that genomic regions that are methylated are preferential sites of recombination. These possibilities are not mutually exclusive and make testable predictions regarding local DNA methylation states prior to and after recombination has taken place.

### Final Thoughts

As the initial report of interference reaches the century mark, some questions regarding how it works are in reach. These questions can be posed within the hypothesis that ZMM proteins stabilize CO intermediates at strand invasion (SEI), which are then committed to an interference-sensitive CO pathway. Does the stabilization of SEI complexes activate a spreading interference ‘signal’ or are CO-designated events evenly spaced to begin with? Do COs occur in two phases, one interfering and the other not? Does Rec8/Spo11 distribution play a role in interference? Does Mus81-Eme1 mediate the resolution of only aberrant recombination intermediates or does it also play a role in mediating a subset of traditional substrates? These questions and many more will have to be answered in order to solve the interference puzzle.

## Figures and Tables

**Table 1:** CO Interference Comparisons Across Model Genetic Organisms. Haploid chromosome number (n) and presence or absence of CO interference is noted. Also shown are presence or absence of Msh4-Msh5 (interference-sensitive) and Mus81-Eme1 (interference-insensitive) mediated CO pathways

Organism	N	Interference?	Msh4-Msh5 COs?	Mus81-Eme1 COs?	~COs/meiosis
*Saccharomyces cerevisiae* [24, 37, 40, 41, 43]	16	Yes	Yes	Yes	90
*Schizosaccharomyces pombe* [48, 89]	3	No	No	Yes	38
*Neurospora crassa* [75, 78, 90]	7	Yes	n.d.	n.d.	20
*Aspergillus nidulans* [91]	8	No	n.d.	n.d.	n.d.
*Caenorhabditis elegans* [92]	6	Yes	Yes	No	6
Arabidopsis thaliana* [44-47, 82, 93]	5	Yes	Yes	Yes	10
*Lycopersicon esculentum* [30]	12	Yes	n.d.	n.d.	21
*Zea mays* [94, 95]	20	Yes	n.d.	n.d.	20
*Drosophila melanogaster* [27, 51, 78]	4	Yes	No	No	6
Danio rerio*[96, 97]	25	Yes	n.d.	n.d.	25-40
Mus musculus*[31, 32, 35, 98-100]	20	Yes	Yes	Yes	22-28
Homo sapiens*[32, 34, 98, 101-106]	23	Yes	Yes	Likely	50-70

n.d. = no data.

* = Reported differences between male and female COs/meiosis.

## ABBREVIATIONS

CO: = Crossover
dHJ: = Double Holliday Junction
DSB: = Double-Strand Break
DSBR: = Double-Strand Break Repair
EN: = Early Nodule
GC: = Gene Conversion
HJ: = Holliday Junction
JM: = Joint Molecule
LN: = Late Nodule
NCO: = Non-Crossover
PMS: = Post-Meiotic Segregation
RN: = Recombination Nodule
SC: = Synaptonemal Complex
SDSA: = Synthesis-Dependant Strand Annealing
SEI: = Single-End Intermediate
ssDNA: = Single-Stranded DNA
ZMM: = *ZIP*, *MSH*, *MER* recombination genes
